# LRPPRC: A Multifunctional Protein Involved in Energy Metabolism and Human Disease

**DOI:** 10.3389/fphys.2019.00595

**Published:** 2019-05-24

**Authors:** Jie Cui, Li Wang, Xiaoyue Ren, Yamin Zhang, Hongyi Zhang

**Affiliations:** ^1^Department of Oncology, The First Affiliated Hospital, Xi'an Medical University, Xi'an, China; ^2^College of General Practitioners, Xi'an Medical University, Xi'an, China; ^3^Department of Urology, The First Affiliated Hospital, Xi'an Medical University, Xi'an, China

**Keywords:** LRPPRC, energy metabolism, mitochondria, LSFC, cancer

## Abstract

The pentatricopeptide repeat (PPR) family plays a major role in RNA stability, regulation, processing, splicing, translation, and editing. Leucine-rich PPR-motif-containing protein (LRPPRC), a member of the PPR family, is a known gene mutation that causes Leigh syndrome French–Canadian. Recently, growing evidence has pointed out that LRPPRC dysregulation is related to various diseases ranging from tumors to viral infections. This review presents available published data on the LRPPRC protein function and its role in tumors and other diseases. As a multi-functional protein, LRPPRC regulates a myriad of biological processes, including energy metabolism and maturation and the export of nuclear mRNA. Overexpression of LRPPRC has been observed in various human tumors and is associated with poor prognosis. Downregulation of LRPPRC inhibits growth and invasion, induces apoptosis, and overcomes drug resistance in tumor cells. In addition, LRPPRC plays a potential role in Parkinson's disease, neurofibromatosis 1, viral infections, and venous thromboembolism. Further investigating these new functions of LRPPRC should provide novel opportunities for a better understanding of its pathological role in diseases from tumors to viral infections and as a potential biomarker and molecular target for disease treatment.

## Introduction

In 1994, Hou J first identified the leucine rich pentatricopeptide repeat (PPR) containing (LRPPRC) gene, which consists of 4.8 kb encoding a 130 kDa product, in HepG2 cells (Ghiso and Lennon, 1994; Hou et al., 1994). Over the past few decades, LRPPRC gene mutation was observed to lead to Leigh syndrome French-Canadian (LSFC), which was caused by a decreased LRPPRC level and steady-state levels of mitochondrial transcripts. LSFC is a genetically homogeneous disease characterized by congenital lactic acidosis. The patients constantly show thrive failure, developmental delay, unique facial appearance and acute crises, including lactic acidosis, hyperglycemia, hepatic cytolysis, and neurological alteration (Mootha et al., 2003; Xu et al., 2004; Debray et al., 2011; Sasarman et al., 2015). In addition, LRPPRC plays a potential role in early onset, multisystem, and neurological presentations of mitochondrial diseases except LSFC (Olahova et al., 2015; Han et al., 2017). Recent studies have reported that LRPPRC dysregulation is related to various diseases ranging from tumors to viral infections. Here, the published data on LRPPRC protein were reviewed. We focused on the functions of LRPPRC, including energy metabolism and maturation and the export of nuclear mRNA, regulatory pathways and its relevance with diseases. The review should provide new opportunities for a better understanding of the role of LRPPRC on human disease and the development of a novel biomarker and treatment in the future.

### Structure of LRPPRC

LRPPRC belongs to the PPR motif-containing proteins family, which comprises a large number of PPR proteins. All PPR proteins contain different numbers of PPR motifs characterized by a degenerate 35-amino-acid motif repeated in tandem, which have been identified in various organisms, ranging from plants to mammals, indicating that PPR is highly conserved during evolution. PPR proteins bind to RNA and regulate transcription, RNA processing, splicing, stability, editing, and translation (Hayes et al., 2012; Manna, 2015).

**Figure 1:**
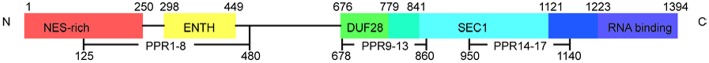
Domain structure of LRPPRC. Phosphorylation sites are located in the 1026–1138 amino acid sequence of the C-terminal domain.

LRPPRC protein contains 23 copies of PPR-, tetratricopeptide- and huntingtin-elongation A subunit-TOR (HEAT)-like tandem repeat sequences. The N-terminus contains multiple copies of leucine-rich nuclear transport signals. The C-terminal sequence contains multiple PPR motifs for RNA-binding (Mili and Pinol-Roma, 2003) and secretory (SEC) 1 structural homology domain for vesicular transport. In addition, LRPPRC contains the Epsin N-terminal homology domain for endocytosis, vesicular trafficking, and cytoskeletal organization change and domain of the unknown function DUF28 homology (Liu and McKeehan, 2001). Phosphorylation sites are located in the 1,026–1,138 amino acid sequence of the C-terminal domain (Ota et al., 2004), suggesting that the interaction of LRPPRC with RNA may be regulated by serine or threonine phosphorylation (Figure 1). The quaternary structure of LRPPRC interacts with the cat eye syndrome chromosome region, candidate 2, heme-binding protein 2, microtubule-associated protein 1S (MAP1S), ubiquitously expressed transcript, peroxisome proliferator-activated receptor gamma coactivator 1 alpha, and forkhead box O1, which are related to energy metabolism (GeneCards, 2019).

### Functions of LRPPRC

LRPPRC is a multifunctional protein that localizes to the outer and inner nuclear membrane, nucleoplasm, endoplasmic reticulum, cytoskeleton, and mitochondria (Liu et al., 2002; Tsuchiya et al., 2004). LRPPRC is identified in multiple protein complexes from mammalian cells and shows no direct activity on their effectors. Instead, LRPPRC plays different roles by direct or indirect protein–protein interactions. The results from STRING database show 25 interacting proteins. DEAD-box helicase 6, cancer susceptibility candidate 3, nuclear cap binding protein subunit 1, interleukin (IL) enhancer-binding factor 3, steroid receptor RNA activator (SRA) stem-loop interacting RNA-binding protein (SLIRP), and LSM4 are related to RNA metabolism. IL-6 and IL-6R are related to acute phase response. Janus kinase 1 (JAK1), JAK2 and JAK3 are related to the activation of non-receptor tyrosine kinase. Cytochrome C, somatic and methyltransferase like 17 are related to the mitochondrial function. Growth factor receptor-bound protein 2, protein tyrosine phosphatase, non-receptor type 11, son of sevenless homolog 1, and (Src homology 2 domain containing) transforming protein 1 participate in the cellular signal pathway. Sec23 homolog A and Ras-related protein Rab-1 A are involved in plasma membrane transport. LSM domain containing protein 1 is an auxiliary component of N-terminal acetyltransferase C. Ubiquitin specific peptidase 19 is a deubiquitinating enzyme that regulates the degradation of various proteins (String, 2019). Thus, the LRPPRC protein is related to multiple cellular functions. The published data available on the LRPPRC protein function are provided in the ensuing sub-sections. However, considering such information, the biological functions and regulatory mechanisms of protein complexes in physiological and pathological conditions are still not fully understood, thus requiring further research.

### LRPPRC and Energy Metabolism

Several studies have identified that the LRPPRC protein plays a specific role in energy metabolism by regulating the mitochondrial DNA (mtDNA)-coded mRNAs but not nuclear-coded mitochondrial mRNAs in human cells (Bangeranye et al., 2010). Mitochondrialtargeting sequence is conserved in mammalian cells. Such sequence exclusively imports to the mitochondrial matrix and cleaves upon entry (Sterky et al., 2010). Similar to other PPR proteins (Herbert et al., 2013), LRPPRC regulates the expressions of the mitochondrial gene at transcriptional and posttranscriptional levels. A reduced expression of LRPPRC occurs after RNA interference (RNAi) transfection interferes with mitochondrial gene transcription and impairs oxygen utilization of cells (Sondheimer et al., 2010a,b). Adeno-associated virus 8 carried shRNA targeting LRPPRC or liver-specific transgenic regulate hepatic LRPPRC level *in vitro* and *in vivo*. LRPPRC promotes fatty acid uptake and oxidation of hepatocytes by increasing oxidative phosphorylation (OXPHOS) activity, which reduces blood lipid level and interdicts non-alcoholic fatty liver disease (NAFLD) in mice. LRPPRC increases OXPHOS activity by associating with mitochondrial RNA polymerase (POLRMT) to form the transcription initiation complex and activate mitochondrial transcription (Liu et al., 2011; Akie et al., 2015; Lei et al., 2016). LRPPRC also binds with peroxisome proliferator-activated receptor coactivator 1-alpha (PGC-1 alpha), a transcriptional coactivator, to regulate the expression of mitochondrial encoded genes, phosphoenolpyruvate carboxykinase (PEPCK), and glucose-6-phosphatase (G6P). A reduction of LRPPRC in fasted mice by adenovirus vector-mediated RNAi inhibits the induction of PEPCK and G6P and blunts hepatic glucose output (Cooper et al., 2006). Similarly, LRPPRC also regulates energy metabolism by interacting with N terminal (NT)-PGC-1 alpha, the splice variant of PGC-1 alpha in brown adipocytes. PGC-1 alpha(–/–) brown adipocytes with NT-PGC-1 alpha expression showed an increased expression of the mtDNA-encoded electron transport chain genes compared with that observed in PGC-1 alpha(–/–) brown adipocytes expressing PGC-1 alpha (Chang and Ha, 2017).

Changes in poly (A) length lead to the dysregulation of the posttranscriptional mitochondrial gene expression and pathogenic defects of respiratory chain complexes. LRPPRC forms a complex with the SLIRP protein, a small SRA binding protein, via an RNA recognition motif–PPR protein interface. The LRPPRC–SLIRP complex is a global RNA chaperone, which binds to mitochondrial mRNAs and stabilizes RNA structures to expose the required sites for polyadenylation and translation by regulating the activity of mitochondrial poly(A) polymerase (Chujo et al., 2012; Ruzzenente et al., 2012; Xu et al., 2012; Lagouge et al., 2015; Spåhr et al., 2016). In addition to the regulatory mechanisms mentioned above, LRPPRC also plays a role in energy metabolism by regulating the activity of ATP synthase. The loss of LRPPRC leads to ATP synthase defect or inactivation of sub-assembled ATP synthase complexes, which contribute to impaired mitochondrial respiration, reduced ATP production, hyperpolarization, and increased mitochondrial reactive oxygen species production in LRPPRC conditional knockout mouse hearts (Mourier et al., 2014). This finding may be caused by the inhibition of the ATPase inhibitory factor 1 mRNA translation by LRPPRC (Esparza-Molto et al., 2018). Consistent with results of studies, the loss of LRPPRC caused an OXPHOS deficiency and decreased the capacity to oxidize fatty acids in LRPPRC conditional knockout mouse livers, which is associated with an ATP synthase complex defect and the loss of cytochrome c oxidase (COX) activity. Livers from 5-week-old LRPPRC hepatocyte-specific knockout mice present loss of lobular organization, steatosis, cholestasis, necrosis/regeneration, and a severe reduction in the COX/succinate dehydrogenase staining ratio (Cuillerier et al., 2015, 2017). Notably, LRPPRC can regulate lipid metabolism in peroxisomes. An analysis of plasma or livers from LSFC patients and LRPPRC hepatocyte-specific knockout mice by a combination of mass spectrometry approaches showed typical characteristics of peroxisomal dysfunction. However, the related mechanism is unknown (Ruiz et al., 2017). Thus, further research is needed. These results indicate that LRPPRC mutation may lead to LSFC or Leigh syndrome through different mechanisms, including COX deficiency, ATP synthase defect, and peroxisomal dysfunction, which may provide an important explanation for the clinical difference between LSFC and other forms of Leigh syndrome.

Mitochondria feature two stress responses to the downregulation of LRPPRC. First, the reduced LRPPRC levels induce mitochondrial hyperfusion, transiently compensate for complex IV activity reduction, and maintain mitochondrial ATP production in mammalian cells. However, prolonged LRPPRC knock-down causes mitochondrial fragmentation and decreased ATP levels (Rolland et al., 2013). Second, the knock-down of LRPPRC in mammalian cells causes an imbalance between nuclear- and mitochondria-encoded subunits of complex IV. The mitochondrial unfolded protein response is triggered by this imbalance for restoration of mitochondrial proteostasis. These distinct pathways are coordinated in response to LRPPRC defects (Kohler et al., 2015). Although maintaining normal ATP levels by compensatory mechanisms, LSFC fibroblasts with reduced LRPPRC show impaired OXPHOS capacity, reduced membrane potential, calcium retention capacity and O_2_ consumption, and increased sensitivity to Ca^2+^-induced permeability transition. Under intense inflammatory or nutritional stresses, such as the tumor necrosis factor or palmitate, the cells present exacerbated death (Bemeur et al., 2012; Burelle et al., 2015; Rafaela et al., 2016). These observations indicate that mitochondria in cells with the LRPPRC defect are dysfunctional, featuring an impaired capacity to maintain energy homeostasis, particularly under inflammatory and nutritional stress conditions. Interestingly, under nutrient overload (palmitate 1 mM and lactate 10 mM), immortalized LSFC fibroblasts become more susceptible to apoptosis and necrosis. Immortalized LSFC cells presented 25% higher citrate synthase activity and 40% lower ATP compared with primary LSFC fibroblasts cells. AMP-activated protein kinase (AMPK) activity decreased in immortalized LSFC cells, suggesting that AMPK activity reduction may compromise the capacity of these cells to palliate energy deficits (Mukaneza et al., 2012; Rivard et al., 2012). Scholars will be interested to determine whether cancer cells with LRPPRC downregulation become more susceptible to apoptosis and necrosis in response to inflammatory and nutritional stresses. Further investigating and elucidating these mechanisms may shed new light on cancer therapy.

Mitophagy is a selective process in which damaged mitochondria is degraded by autophagy to maintain energy homeostasis. This process plays an important role in cell protection. Phosphatase and tensin homolog (PTEN)-induced putative kinase 1 (PINK1)–Parkin pathway is a crucial mechanism that triggers mitophagy (Durcan and Fon, 2015; Pickrell and Youle, 2015). LRPPRC interacts with Parkin and stabilizes Parkin substrates, including Bcl-2 and Parkin itself, to inhibit autophagy (Zou et al., 2014). Moreover, LRPPRC forms a ternary complex with Beclin 1 and Bcl-2. Reduced LRPPRC causes a reduction of Bcl-2 due to Parkin degradation. More Beclin1 is released to form Beclin 1–class III phosphatidylinositol 3-kinase (PI3K) complex. Autophagy is triggered to accelerate the degradation of dysfunctional mitochondria through the PI3K/AKT/mammalian target of the rapamycin (mTOR) pathway (Zou et al., 2013, 2015). Numerous studies have demonstrated that mitophagy dysregulation causes neurodegeneration (Shefa et al., 2019). Further studies are needed to investigate whether LRPPRC plays an important role in LSFC through mitophagy dysregulation. In summary, LRPPRC regulates energy metabolism through multiple mechanisms. Oxidative stress plays significant roles in diverse pathophysiological processes, including neurodegenerative diseases and cancer (Thanan et al., 2014). Further investigating and elucidating these mechanisms may provide a therapeutic target to mitigate certain metabolic disorders.

### LRPPRC and Maturation and Export of Nuclear mRNA

LRPPRC associates with heterogeneous nuclear ribonucleoprotein A1-associated poly(A) mRNAs and is a part of nuclear mRNPs complexes involved in the maturation and export of nuclear mRNA (Mili et al., 2001; Tsuchiya et al., 2004). Eukaryotic initiation factor 4E (eIF4E), the mRNA 5′ cap-binding protein, plays a role in translational control through its effects on mRNA export and cap-dependent translation, both of which contribute to oncogenic potential. The mRNA export of eIF4E-sensitivity element (4E-SE)-containing mRNAs is positively regulated in an eIF4E-dependent manner. LRPPRC is identified to be a cofactor of eIF4E mRNA export by an RNP isolation/mass spectrometry approach. The protein binds with mRNAs containing 4E-SE and alters several eIF4E-sensitive mRNA export functions (Topisirovic et al., 2009; Culjkovic et al., 2013). LRPPRC directly binds to chromosome maintenance protein 1, which possibly acts as an export receptor, and allows the eIF4E-LRPPRC-4ESE RNA complex to transport from nucleus into cytoplasm. Moreover, importin 8, the nuclear importer of cap-free eIF4E, imports RNA-free LRPPRC to increase the efficiency of future export cycles (Volpon et al., 2017). Thus, we suggest that LRPPRC may play an important role in tumorigenesis by connecting an oncogenic gene expression with energy production.

### LRPPRC Regulatory Pathways

**Figure 2:**
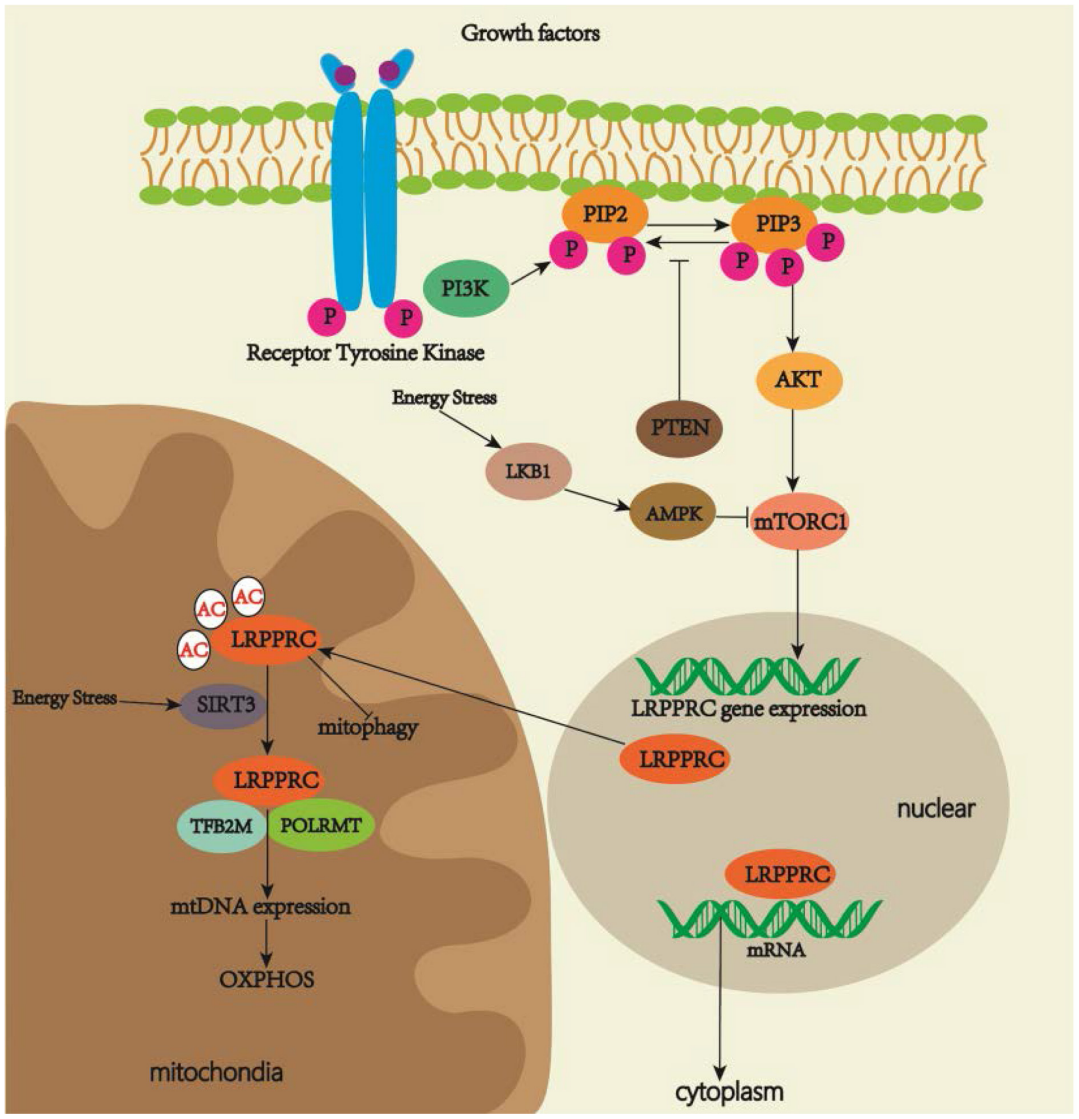
Regulatory pathways of LRPPRC. LRPPRC plays key roles in regulating OXPHOS activity, mitophagy and maturation and export of nuclear mRNA. MTORC1 activation increases the expression of LRPPRC. Energy stress increases SIRT3 activity, which promotes deacetylation of LRPPRC for increased OXPHOS activity.

Several top predicted transcription factors for regulating the LRPPRC gene expression include the signal transducers and activators of the transcription (STAT) family, which is related to development, differentiation, cell proliferation and survival, and NK2 homeobox 5, which is involved in congenital heart disease (GeneCards, 2019). The luciferase reporter gene and chromatin immunoprecipitation assay will be necessary to study interactions between multiple specific STAT family members and promoter sequences of LRPPRC. Current studies on LRPPRC signal pathways are limited. PI3K/AKT/mTOR signaling is an evolutionarily conserved pathway that regulates cell proliferation and growth (LoRusso, 2016). MTOR plays a role in mitochondrial function by regulating mitochondrial gene transcription and translation (Wei et al., 2015). MTOR complex 1 (mTORC1) inhibition with rapamycin decreases LRPPRC expression, which is related to the selective reduction of COX expression in LSFC fibroblasts. These results demonstrate that PI3K/AKT/mTOR signaling may play a role in OXPHOS activity by regulating the LRPPRC expression (Mukaneza et al., 2017). As LRPPRC contains phosphorylation sites (Ota et al., 2004), determining whether mTOR regulates OXPHOS activity through the direct phosphorylation of LRPPRC should be of scientific interest. Another study has revealed that nutrient deprivation induces sirtuin 3 (SIRT3), NAD-dependent deacylases, and ADP-ribosyltransferases, all of which are critical in the response to mitochondrial stress (Yang et al., 2016). Glucagon activates cAMP signaling and increases SIRT3 activity in the liver, thus regulating lysine residues to generate a lysine-to-arginine mutant of LRPPRC that could be identified by mass spectrometry. The mutant increases mitochondrial transcription and OXPHOS activity by strengthening LRPPRC–POLRMT interactions in the liver mitochondria of fasted mice. Increased OXPHOS activity augments fatty acid oxidation (FAO), gluconeogenesis, ketogenesis, and ureagenesis (Liu et al., 2014), thus suggesting that LRPPRC plays a role in the machinery of mitochondrial response under nutrient deprivation (Figure 2). We suggest that energy stress leads to mTORC1 inhibition (Schneider et al., 2008) and a decreased expression of LRPPRC. On the one hand, the downregulation of LRPPRC reduces the export of eIF4E-sensitive transcripts for cell metabolism and growth, and triggers autophagy to accelerate the degradation of dysfunctional mitochondria for cell protection. On the other hand, LRPPRC in mitochondria is deacetylated by SIRT3, which strengthens LRPPRC–POLMAT interactions for mtDNA expression and increases OXPHOS activity in response to energy deficits.

### LRPPRC and Human Disease

#### LRPPRC and Tumors

Several studies have shown that LRPPRC expression increases in various cancer tissues and cell lines, including prostate cancer (PCa) (Jiang et al., 2014, 2015; Zhang H. Y. et al., 2017), gastric cancer (Tian et al., 2012; Li X. et al., 2014; Gao et al., 2015), lung adenocarcinoma (Tian et al., 2012; Fahrmann et al., 2016), esophageal squamous cell carcinoma (Tian et al., 2012), colon cancer (Tian et al., 2012; Nishio et al., 2017), mammary and endometrial adenocarcinoma (Tian et al., 2012), and lymphoma (Tian et al., 2012); by contrast, normal tissues hardly or lowly express LRPPRC (Table 1). The LRPPRC level is positively associated with tumor grade, metastasis, and serum prostate-specific antigen levels in patients with PCa but negatively associated with biochemical progression-free and overall survival (Jiang et al., 2014; Zhang H. Y. et al., 2017). A number of investigations have determined that high LRPPRC combined with low MAP1s is significantly related to poor prognosis. MAP1s links mitochondria with microtubules for trafficking and affects autophagosomal biogenesis and degradation, thereby increasing autophagy and suppressing tumorigenesis (Jiang et al., 2015); these findings suggest that LRPPRC may contribute to autophagy inhibition to increase the tumorigenesis of PCa. Ahigh expression of LRPPRC has been consistently and negatively associated with overall survival in patients with gastric cancer (Li X. et al., 2014). Several potential biomarkers for mitochondrial OXPHOS and FAO are also increased in gastric cancer tissues (Gao et al., 2015), thus suggesting that LRPPRC may play a role in the progression of gastric cancer by promoting OXPHOS and FAO activity. Proteomics profiling has shown that increased LRPPRC could be identified in current or former smokers with early-stage (Stage IA/IB) lung adenocarcinoma, which suggests that the dysregulation of the protein may initiate carcinogenesis (Fahrmann et al., 2016). LRPPRC combined with Ral guanine nucleotide dissociation stimulator (RALGDS), LIM and SH3 protein 1 (LASP1), glucose-6-phosphate dehydrogenase (G6PD), ADRBKI, and proteasome subunit alpha 1 (PSMA1) has been significantly associated with an increased risk of relapse of chronic myeloid leukemia (Oehler et al., 2010). These results demonstrate that LRPPRC overexpression is strongly associated with disease progression and poor prognosis. Interestingly, the promoter sequences of LRPPRC have been reported to be hypermethylated in squamous cell carcinoma of the tongue, although the pathological significance is currently unknown (Bhat et al., 2017). These observations suggest that the role of LRPPRC in tumors depends on the specific tumor type. Thus, LRPPRC may act as a diagnostic and prognostic tumor marker. As normal cells hardly or lowly express LRPPRC, the protein may be a potential molecular target for cancer therapy.

**Table 1:** Expression of LRPPRC in various tumor types.

**Author**	**Cancer samples**	**Method**	**LRPPRC expression**
Oehler et al., 2010	213 bone marrow samples from chronic myeloid leukemia patients	Quantitative PCR	The model including RALGDS, LASP1, G6PD, ADRBKI, LRPPRC and PSMA1 is significantly associated with an increased risk of relapse
Fahrmann et al., 2016	38 lung adenocarcinomas	Proteomics approach and immunohistochemistry	LRPPRC levels are increased in cancer tissues compared with non-malignant paired tissues
Li X. et al., 2014	253 gastric cancers	Immunohistochemical staining	LRPPRC levels are increased in cancer tissues compared with paired control tissues
Gao et al., 2015	12 gastric cancers	Tandem mass tag (TMT) method followed by mass spectrometry	LRPPRC levels are increased in cancer tissues compared with adjacent normal tissues
Jiang et al., 2014	112 prostate cancers	Immunohistochemical staining	LRPPRC levels are increased in cancer tissues compared with BPH tissues
Jiang et al., 2015	111 prostate cancers	Immunohistochemical staining	LRPPRC levels are increased in cancer tissues compared with BPH tissues
Zhang H. Y. et al., 2017	198 prostate cancers	Immunohistochemical staining	LRPPRC levels are increased in cancer tissues compared with BPH tissues
Nishio et al., 2017	133 colorectal cancers	Isobaric tags for relative and absolute quantitation (iTRAQ) and immunohistochemistry	LRPPRC levels are increased in cancer tissues compared with normal colorectal tissues
Bhat et al., 2017	20 squamous cell carcinomas of the tongue	Differential methylation hybridization (DMH) microarray and bisulphite genome sequencing (BGS)	Gene promoter-associated CpG islands of LRPPRC are hypermethylated
Tian et al., 2012	20 lung adenocarcinomas, 9 esophageal squamous cell carcinomas, 14 gastric cancers, 7 colon cancers, 11 mammary and 13 endometrial adenocarcinomas, 8 diffuse large B-cell lymphomas	Immunohistochemical staining	Cancer tissues express LRPPRC abundantly, whereas the surrounding non-neoplastic cells hardly express LRPPRC

The consequences of aberrant LRPPRC expression have been investigated. Downregulation of LRPPRC by LRPPRC siRNA inhibits the growth of gastric cancer cells (Li X. et al., 2014). In another study, the knock-down of the LRPPRC expression reduces anti-apoptosis, invasion, and *in vitro* colony-forming capacities of lung adenocarcinoma and Hodgkin lymphoma cells; such condition is related to the inhibition of pro-survival Bcl-2 family members (Tian et al., 2012). Downregulation of LRPPRC by siRNA consistently reduces the invasion capacity and promotes apoptosis of PCa cells through the mitochondrion-mediated pathway (Zhou et al., 2014; Zhang H. et al., 2017). These observations suggest that LRPPRC plays an important role in apoptosis resistance and enhances the invasion capacity of tumor cells. As an elevated LRPPRC was observed in PCa tissues isolated from PTEN–/– mice by immuno-fluorescence staining (Jiang et al., 2014), determining whether dysregulation of the PTEN/PI3K/AKT/mTOR signaling pathway leads to oncogenesis of PCa by increasing LRPPRC expression would be worth studying.

The “Warburg effect” is an important feature of cancer cells and refers to the fermentation of glucose to lactate in the presence of oxygen instead of complete oxidation of the former to fuel mitochondrial respiration. We now understand that the Warburg effect is not the only route to ATP production in cancer cells. In specific cancer cells, mitochondrial respiration remains intact; thus, several cancer subtypes mainly depend on OXPHOS activity (Vyas et al., 2016; Zhu et al., 2018). FAO is one of the pathways through which cancer cells gain energy. Tumors, such as diffuse large B-cell lymphoma and PCa, are highly dependent on FAO for survival and growth (Qu et al., 2016). Fatty acid synthase (FASN) overexpression has been regarded as an early event in prostate tumorigenesis and is associated with cancer progression; several inhibitors of FASN have been demonstrated to inhibit proliferation and induce the apoptosis of PCa cells (Twum-Ampofo et al., 2016). Thus, determining whether elevated LRPPRC provides more energy for cancer cells by promoting OXPHOS and FAO activity would be of scientific interest.

P-glycoprotein (Pgp), which is encoded by the ATP-binding cassette sub-family B member 1 (ABCB1) gene, acts as an efflux pump and contributes to multidrug resistance (MDR). The relation between LRPPRC and MDR has been investigated in a number of tumors. Proteomics has determined that chronic myeloid leukemia MDR/imatinib mesylate (IM) cross-resistant cells express higher levels of LRPPRC mRNA and protein compared with their parental cells, thus suggesting that LRPPRC could be used as a putative actor in IM resistance and predict therapy responses (Corrêa et al., 2010, 2012). Further investigation has identified that LRPPRC acts as a transcription factor for ABCB1 expression via an invMED1 binding site in ABCB1. Regulation through LRPPRC is affected by the methylation status of the GC-100 box in the ABCB1 promoter (Labialle et al., 2004; Correa et al., 2014). Another study showed the overexpression of LRPPRC in MDR gastric cancer cells. Cytotoxic drug sensitivity is significantly enhanced by the downregulation of LRPPRC, and the capacity of the transporter protein Pgp to efflux adriamycin is reduced, which is related to decreased MDR-1 transcriptional activity (Li X. S. et al., 2014). However, the capacity of hepatocarcinoma cells to extrude drugs is unaffected by the protein as decreased LRPPRC expression is insufficient to reduce Pgp production (Michaud et al., 2011); this finding actually contradicts the aforementioned studies and could be explained by the supposition that the role of LRPPRC in the MDR phenotype depends on the specific type of cancer. Overall, however, the results indicate that LRPPRC may serve as a potential molecular target for MDR reversal.

#### Potential Role of LRPPRC in Other Diseases

##### LRPPRC and Parkinson's disease (PD)

PD is a neurodegenerative disorder (Joers et al., 2017) associated with defects in the Parkin/PINK1 pathway for mitochondrial quality control (Kim et al., 2012; Gaweda-Walerych and Zekanowski, 2013). LRPPRC, in cooperation with Parkin, plays a potential role in PD pathogenesis (Gaweda-Walerych and Zekanowski, 2013). LRPPRC is severely decreased in PINK1 dopaminergic neuronal null cells, and the overexpression of LRPPRC augments complex IV activity, thus suggesting that PINK1 regulates complex IV activity through interactions with LRPPRC (Kim et al., 2012). Dedicated minigene assays for PD-related genetic variants show that LRPPRC intronic variants influence pre-messenger RNA splicing by regulating the inclusion of corresponding exons, and this event is associated with the risk of PD and related disorders (Gaweda-Walerych et al., 2016). These results demonstrate that LRPPRC plays a potential role in the pathogenesis of PD and provides a potential therapeutic target for the disease. The development of appropriate animal models will bear importance in understanding the role of LRPPRC in PD progression.

##### LRPPRC and neurofibromatosis 1 (NF1)

NF1, with a birth incidence of 1:3,000, is the most common inherited tumor-predisposing syndrome in humans caused by a loss of function mutations or NF1 gene deletions. By constructing a number of NF1-GST domains combined with differential mass spectroscopic analysis and immunoprecipitation, the NF1-tubulin binding domain was identified to interact with the LRPPRC protein, which occurs predominantly along microtubules and complexes with motor proteins. This interaction links NF-1 with LSFC (Arun et al., 2010a,b, 2013). Further investigation of the importance of this novel interaction contributing to the manifestations of NF-1 and LSFC is expected to yield interesting findings.

##### LRPPRC and viral infection

LRPPRC plays a role in viral infection. One study revealed that LRPPRC is associated with the human immunodeficiency virus (HIV)-1 nucleic acids during the early steps of viral infection through co-immunoprecipitation and reverse transcription-PCR experiments. A knock-down of LRPPRC reduces HIV-1 replication in cell lines but shows no influence on viral production and RNA encapsidation (Schweitzer et al., 2012). Another study revealed that the downregulation of LRPPRC produces a significant inhibition of the HCV infection. LRPPRC acts as a nonstructural protein 5A (NS5A) binding factor. NS5A contributes to the inhibition of innate immunity during the hepatitis C viral infection by exploiting the capacity of LRPPRC to inhibit the antiviral signals regulated by the mitochondrial antiviral-signaling protein (Refolo et al., 2019). These observations suggest that LRPPRC plays a crucial role in antiviral ability and provides a potential therapeutic target for the therapy of viral diseases. However, the related mechanisms are still not fully understood. Future examination of mitochondrial dysfunction mediated by the LRPPRC and gene array analysis of cells with LRPPRC downregulation during viral infection may reveal novel pathways that are critical for such infection.

##### LRPPRC and venous thromboembolism (VTE)

VTE is associated with atherosclerosis and the risk of arterial cardiovascular events (Sorensen et al., 2007). Although a strong heritability component is observed, only half of all patients present established genetic risk factors. Whole-exome sequencing in two thrombophilic families showed that the variant of LRPPRC (LRPPRC rs372371276) remains a putative disease-risk candidate of VTE (Cunha et al., 2015, 2017).

## Conclusions

Over the past 20 years, considerable progress has been achieved in understanding the functions of LRPPRC, which is involved in energy and mRNA metabolism. As the protein is involved in mRNA stability, determining whether long non-coding or small RNAs are regulated by LRPPRC would be of interest. LRPPRC has been implicated in human diseases involving Leigh syndrome, LSFC, cancer, PD, NF1, viral infection, VTE, and NAFLD. These novel findings provide new insights into the physiological and pathological functions of the protein. However, the molecular mechanisms activated in these diseases are not fully understood. Moreover, the signal pathway of LRPPRC is largely unknown. PI3K/AKT/mTOR and JAK/STAT pathway dysregulation may play important roles in human diseases through LRPPRC dysfunction, and this supposition requires further investigation. Therefore, understanding the precise function and role of LRPPRC and protein–protein complexes in physiological and pathophysiological conditions bears importance. Targeting LRPPRC to either increase or decrease its function under different pathological states could be a novel treatment approach for various diseases.

The LRPPRC expression has been shown to increase in multiple types of tumors and is associated with poor prognosis and resistance. The downregulation of LRPPRC induces apoptosis and reduces the invasion capacity of cancer cells, thus suggesting that LRPPRC may serve as a promising biomarker and potential molecular target. Despite these advances, however, research on the role of LRPPRC in tumors remains limited, and several basic questions remain unanswered. For example, whether subcellular localization of the protein determines its different effects on tumorigenesis and progression is unclear. Furthermore, whether the downregulation of LRPPRC causes cancer cells to become more susceptible to apoptosis inducers under inflammatory and nutritional stress, which would be useful for therapeutic development, is unknown. Answers to these questions may provide an important foundation for the understanding of the pathological role of LRPPRC in tumors and advance efforts to develop a potential biomarker and molecular target for tumor treatment.

## Author Contributions

JC, HZ, and YZ collected references and wrote the manuscript. LW and XR also collected references.

### Conflict of Interest Statement

The authors declare that the research was conducted in the absence of any commercial or financial relationships that could be construed as a potential conflict of interest.

## Abbreviations

PPR: pentatricopeptide repeat
TPR: tetratricopeptide
HEAT: huntingtin-elongation A subunit-TOR
SEC1: secretory 1
ENTH: Epsin N-terminal Homology
DUF: Domain of Unknown Function
LSFC: Leigh syndrome French-Canadian
mtDNA: mitochondrial DNA
ATP: adenosine triphosphate
POLRMT: mitochondrial RNA polymerase
PGC-1 alpha: peroxisome proliferator-activated receptor coactivator 1-alpha
COX: cytochrome c oxidase
ETC: electron transport chain
SRA: steroid receptor RNA Activator
mtPAP: mitochondrial poly(A) polymerase
OXPHOS: oxidative phosphorylation
PTP: permeability transition pore
UPR mt: mitochondrial unfolded protein response
mRNP: messenger ribonucleo-protein
NE: nuclear envelope
ER: endoplasmic reticulum
EIF4E: Eukaryotic initiation factor 4E
4E-SE: eIF4E-sensitivity element
CRM1: chromosome maintenance protein 1
PINK1: PTEN induced putative kinase 1
OMM: outer mitochondrial membrane
PCa: prostate cancer
BPH: benign prostatic hyperplasia
FAO: fatty acid oxidation
PEPCK: phosphoenolpyruvate carboxykinase
G6P: glucose-6-phosphatase
SIRT 3: sirtuin 3
BCP: biochemical progression
FASN: fatty acid synthase
Pgp: P-glycoprotein
MDR: multidrug resistance
CML: chronic myeloid leukemia
IM: imatinib mesylate
PD: Parkinson's disease
DA: dopaminergic
SN: substantia nigra
NF1: neurofibromatosis 1
TBD: tubulin binding domain
VTE: venous thromboembolism.
